# Context-dependent NMDA receptor dysfunction predicts seizure treatment in mice with human GluN1 variant

**DOI:** 10.1016/j.isci.2025.114301

**Published:** 2025-12-01

**Authors:** Sridevi Venkatesan, Daria Nazarkina, Megan T. Sullivan, Yao-Fang Tan, Sarah Qu, Amy J. Ramsey, Evelyn K. Lambe

**Affiliations:** 1 Department of Physiology, Temerty Faculty of Medicine, University of Toronto, Toronto, ON M5S 1A8, Canada; 2 Hospital for Sick Children, Toronto, ON M5G 0A4, Canada; 3 Department of Pharmacology and Toxicology, Temerty Faculty of Medicine, University of Toronto, Toronto, ON M5G 2C8, Canada; 4 Department of OBGYN, Temerty Faculty of Medicine, University of Toronto, Toronto, ON M5G 1E2, Canada; 5 Department of Psychiatry, Temerty Faculty of Medicine, University of Toronto, Toronto, ON M5S 1A1, Canada

**Keywords:** genetics, neuroscience

## Abstract

Mutations in N-methyl-D-aspartate receptors (NMDARs) cause epilepsy and profound cognitive impairment, though the underlying subunit-specific vulnerabilities remain unclear. We investigate the impact of a severe human variant in the lurcher motif of obligate GluN1 NMDAR subunit using transgenic mice, revealing unexpected context-dependent phenotypes. We show that the GluN1 Y647S variant significantly reduces current flow through pharmacologically isolated synaptic NMDARs in prefrontal neurons. Yet in intact local circuits, this loss-of-function paradoxically extends NMDAR-dependent dendritic integration, causing prolonged circuit-wide excitation that promotes seizures. Mutant receptors appear deficient in engaging opposing dendritic ion channels that normally curtail NMDAR-dependent excitation. Boosting SK channel activity normalizes dendritic integration, whereas slight decreases in extracellular magnesium further extend abnormally prolonged integration in mutant mice. We find that magnesium supplementation successfully treats seizures *in vivo* in the transgenic mice, despite loss-of-function of NMDARs. Overall, we disentangle a GluN1 variant’s receptor-level effects and its dendritic impact to treat seizures effectively.

## Highlights

• GluN1 Y647S^+/−^ variant reduces NMDA receptor function in mouse prefrontal cortex
• Despite reduced receptor activity, dendritic excitation is prolonged and triggers seizures
• Sensitizing SK channels restores magnesium blockade of NMDA receptors
• Oral magnesium threonate prevents seizures in mice with the Y647S^+/−^ variant

## Graphical abstract

**Figure:**
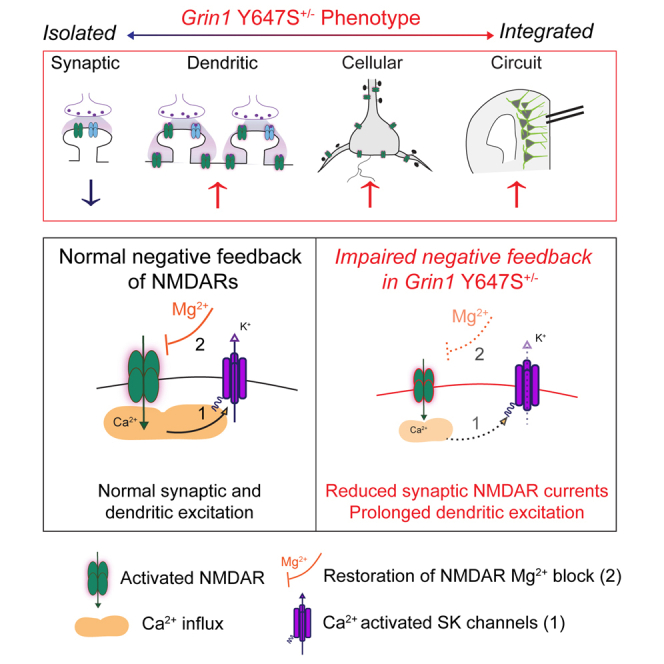

## Introduction

N-methyl-D-aspartate receptors (NMDARs) are heterotetrametric ligand gated ion channels activated by the excitatory neurotransmitter glutamate.^1^^,^^2^ NMDARs are vital for normal brain development and physiology and their disruption is implicated in multiple neurobiological disorders.^3^^,^^4^^,^^5^ *De novo* missense mutations in the *GRIN* family of genes encoding for different NMDAR subunits cause a rare neurodevelopmental disorder characterized by epilepsy, developmental delay, and intellectual disability.^6^^,^^7^^,^^8^

NMDAR patient variants have been previously examined using heterologous expression systems including oocytes,^9^^,^^10^^,^^11^^,^^12^ HEK cells,^13^^,^^14^^,^^15^ or viral expression in brain slice cultures.^16^ These approaches characterize mutant NMDARs in artificial environments, classifying variants as loss- or gain-of-function based on the ion channel properties. It is increasingly becoming evident that comprehensive assessment in intact neural circuits using transgenic mice is further necessary to understand and treat the neurological consequences of NMDAR variants.^15^^,^^16^^,^^17^^,^^18^^,^^19^^,^^20^ Particularly, human variants in the obligate GluN1 NMDAR subunit that cause severe neurological deficits in patients^8^^,^^21^ are yet to be examined.

The Y647S substitution occurs in a highly conserved transmembrane region of the NMDA receptor (NMDAR) known as the lurcher motif^22^ and causes severe epilepsy and developmental delay in the patient.^21^ Heterologous expression studies of NMDARs with this mutation have revealed reduced surface expression as well as reduced open channel probability and variable changes in agonist binding.^6^^,^^21^^,^^23^^,^^24^ These reports are unclear on whether this results in clear loss or gain-of-function of the receptor. Transgenic heterozygous *Grin1* Y647S^+/−^ mice carrying this variant were recently generated and mimic the patient phenotype, providing an opportunity to evaluate NMDAR function in intact circuits.^25^

We focus on NMDARs in the prefrontal cortex, since they play a critical role in higher order cognition by driving dendritic integration that is essential for healthy brain function.^26^^,^^27^^,^^28^^,^^29^^,^^30^ Genetic disruptions to NMDARs can result in heterogeneous deficits across subcellular compartments, rendering dendritic integration highly vulnerable.^31^^,^^32^ Therefore, we examined the context-specific effects of the *Grin1* Y647S variant in the medial prefrontal cortex.

By assessing endogenous glutamate-mediated responses, we find that *Grin1* Y647S^+/−^ neurons have reduced isolated, synaptic NMDAR currents yet display paradoxically prolonged NMDAR-dependent dendritic integration. This is accompanied by protracted Ca^2+^ influx that triggers seizure-like events. Boosting SK channel activity is sufficient to prevent dendritic hyperexcitation and restore normal dendritic kinetics, suggesting that mutant NMDARs may insufficiently engage typical negative feedback via Ca^2+^ activated potassium channels.^33^ Furthermore, Y647S^+/−^ neurons are extremely sensitive to extracellular Mg^2+^ and show excessive NMDAR-dependent currents under low Mg^2+^ conditions. Therefore, we treat *Grin1* Y647S^+/−^ mice with oral magnesium L-threonate (MgT) supplementation, which has been shown to boost brain Mg^2+,^^34^^,^^35^ observing significant decreases in seizure occurrence and severity.

Our work reveals an unexpected mechanism for epilepsy caused by a loss-of-function *GRIN1* variant and highlights dietary magnesium supplementation as an effective treatment. We identify context-dependent effects of a genetic NMDAR variant that provide mechanistic insights for future treatment strategies.

## Results

**Figure 1:**
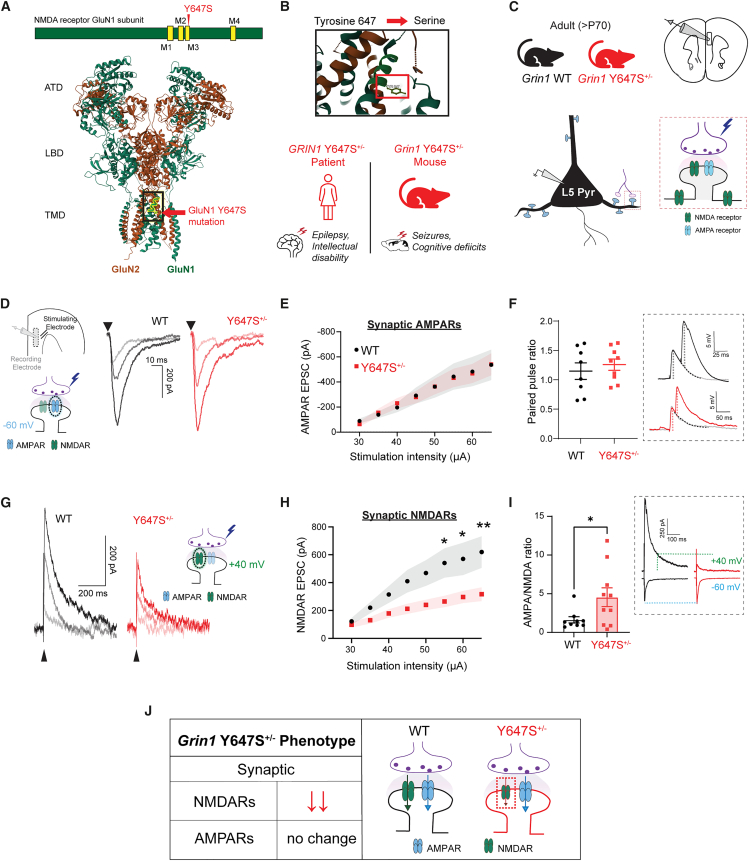
Isolated NMDARs show loss of function in mice with GluN1 Y647S^+/−^ patient variant (A) Schematic showing the position of the Y647S mutation in the M3 transmembrane domain of the GluN1 NMDAR subunit (top), structure of the human GluN1-GluN2A NMDAR tetramer (PDB: 6IRF)^36^ highlighting the Y647 site in the channel pore (bottom). (B) Tyrosine residue at the 647^th^ position of GluN1 is mutated to serine, causing severe seizures and intellectual disability in the patient. Mutant mice (*Grin1* Y647S^+/−^) replicating the patient phenotype were used for slice electrophysiology. (C) Coronal prefrontal cortex slices from adult WT and mutant (Y647S^+/−^) mice were used to record synaptic currents evoked by electrical stimulation of glutamate release in layer 5 pyramidal (L5 Pyr) neurons. (D) Schematic with position of stimulating and recording electrodes in the brain slice. Isolated AMPAR EPSCs measured using cesium gluconate patch pipettes in the presence of GABAR blockers at −60 mV in WT and Y647S^+/−^ at increasing stimulus strengths. (E) AMPAR EPSC peak amplitude in WT and Y647S^+/−^ at different stimuli is identical*.* (F) Paired pulse ratio at 20 ms interval is similar in WT and Y647S^+/−^ neurons*.* (G) Isolated NMDAR EPSCs measured with AMPARs blocked at +40 mV in WT and Y647S^+/−^. (H) NMDAR EPSC peak amplitude is reduced in Y647S^+/−^ neurons (∗*p* < 0.05, ∗∗*p* < 0.01, Sidak’s post hoc). (I) AMPA to NMDA ratio in WT and Y647S^+/−^ neurons (∗*p* < 0.05, unpaired *t* test). (Inset), EPSCs at −60 and +40 mV in WT and Y647S^+/−^, dotted line indicates NMDAR component at +40 mV. (J) Summary schematic showing preserved AMPAR but reduced NMDAR currents in *Grin1* Y647S^+/−^ mice. Bars denote mean ± SEM.

We investigate the neurophysiological consequences of the Y647S *GRIN1* variant in the transmembrane region of the GluN1 NMDAR subunit that causes intellectual disability and epilepsy^6^^,^^7^^,^^21^ in a patient. Using a mouse model (*Grin1* Y647S^+/−^ mice) that replicates patient phenotypes of epilepsy and cognitive deficits,^25^ we examine prefrontal neurotransmission and NMDAR signaling to decipher and treat underlying disease mechanisms (Figures 1A–1C).

### Impact on prefrontal glutamate synapses

#### Intact AMPAR transmission in *Grin1* Y647S^+/−^ mice

To determine the effect of the *Grin1* Y647S variant on glutamatergic synapses in intact neural circuits, we measured excitatory postsynaptic currents (EPSCs) in principle neurons in response to electrically evoked glutamate release. Using experimental conditions designed to strictly isolate glutamatergic synaptic currents in prefrontal brain slices (see evoked EPSCs under STAR Methods), we found similar AMPA receptor (AMPAR) EPSC amplitudes between WT and Y647S^+/−^ mice (Figures 1D–1E; F_(1, 143)_ = 0.001, *p* = 0.98, *N* = 7, 8 mice, respectively).

AMPAR postsynaptic potentials and paired pulse ratio of postsynaptic potentials further showed no difference between WT and Y647S^+/−^ neurons (Figure 1F; *t*_(15)_ = 0.66, *p* = 0.52). Average miniature EPSC amplitude was similar in WT (13 ± 2 pA) and Y647S^+/−^ (16 ± 2 pA, *t*_(12)_ = 0.07, *p* = 0.94). Average miniature EPSC frequency also did not show significant changes between WT (9 ± 1 Hz) and Y647S^+/−^ (9 ± 2 Hz, *t*_(12)_ = 1.21, *p* = 0.25) neurons. We conclude that presynaptic glutamate release probability and postsynaptic AMPAR strength are not altered by the Y647S variant.

#### Loss of function of NMDARs in *Grin1* Y647S^+/−^ mice

Next, we directly assessed postsynaptic current through NMDARs under experimental conditions that isolate NMDAR glutamatergic synaptic currents in prefrontal brain slices. Previous biophysical examination indicates that the GluN1-Y647S variant drastically reduces open channel probability (83% reduction) and surface expression (84% reduction), leading to decreased functional activity^25^ in heterologous expression systems despite the variant’s enhanced affinity for glutamate.^25^

Consistently, we found that the amplitude of isolated synaptic NMDAR EPSCs was substantially reduced in Y647S^+/−^ neurons, with 50% reduction in peak amplitude at the highest tested stimulus (Figures 1G and 1H; two-way ANOVA, genotype: F_(1, 192)_ = 37.78, *p* < 10^−4^; *t*_(192)_ = 3.11, ∗*p* = 0.017, Sidak’s post hoc). Reduction in NMDAR EPSC amplitude was observed in both sexes, with no significant effect of sex (F_(1, 22)_ = 0.005, *p* = 0.94). In addition to reduced amplitude, Y647S^+/−^ NMDAR EPSCs also showed significantly faster decay kinetics with reduced half-width (WT: 94 ± 8 ms, Y647S^+/−^: 64 ± 8 ms, *t*_(16)_ = 2.64, *p* = 0.018). D-AP5 was equally effective at blocking NMDARs in both genotypes (two-way repeated measures ANOVA, D-AP5: F_(1, 10)_ = 49.99, *p* < 10^−4^) indicating similar receptor composition and antagonist efficacy.

To evaluate the relative loss of NMDAR function with respect to AMPARs within the same cell, we calculated AMPA/NMDA ratio (Figure 1I, inset). Consistent with the preservation of AMPAR but reduction in NMDAR responses, Y647S^+/−^ neurons showed significantly increased AMPA/NMDA ratio (WT: 1.6 ± 0.4, Y647S^+/−^ = 4.5 ± 1.2, *t*_(17)_ = 2.18, *p* = 0.04). In summary, the GluN1 Y647S variant results in loss of synaptic NMDAR currents in excitatory neurons without altering AMPARs or glutamate release probability (Figure 1J).

### Impact on integrative NMDAR signaling

#### Paradoxically prolonged NMDAR plateau potentials in *Grin1* Y647S^+/−^ mice

**Figure 2:**
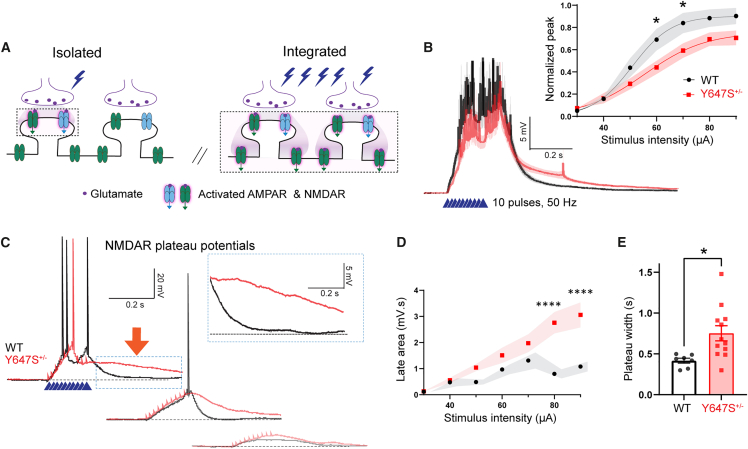
Paradoxically prolonged NMDAR integration in *Grin1* Y647S^+/−^ mice (A) Schematic showing that a single stimulus evokes limited glutamate release that activates only synaptic receptors, while repetitive high frequency stimulation causes glutamate spillover onto the dendrite, generating a dendritic plateau potential. (B) Average NMDAR plateau potentials in WT and Y647S^+/−^ neurons evoked by 50 Hz stimulation, measured in the presence of AMPAR and GABAR blockers. (Inset) Plateau potential amplitude (normalized to the spike threshold) at different stimulus intensities in WT and Y647S^+/−^ (∗*p* < 0.05, Sidak’s post hoc). (C) NMDAR plateau potentials show extended duration in Y647S^+/−^ neurons compared to WT at multiple stimulus intensities. (D) Area under the plateau potential for the 1 s period post-stimulation (late area) at different stimuli in WT and Y647S^+/−^ (∗∗∗∗*p* < 10^−4^, Sidak’s post hoc). (E) Total duration of the plateau potential (at 50 μA stimulation) in WT and Y647S^+/−^ neurons (∗*p* < 0.05, unpaired *t* test).

To determine the impact of the Y647S mutation on integrative NMDAR signaling, we examined NMDAR plateau potentials in prefrontal brain slices under typical whole-cell recording conditions (K+ gluconate pipette, −75 mV current clamp) with AMPAR and GABAR blockers. Plateau potentials are NMDAR-dependent dendritic phenomena caused by glutamate spillover during repetitive stimulation and are vital for cognitive integration^37^^,^^38^ (Figure 2A). NMDAR plateau potentials elicited by 50 Hz stimulation (10 pulses) at basal dendrites and recorded at the cell body showed a significant reduction in peak amplitude in Y647S^+/−^ neurons (Figure 2B; two-way ANOVA, genotype: F_(1, 266)_ = 17.95, *p* < 10^−4^, *N* = 10, 12 mice for WT and Y647S^+/−^). This decrease in Y647S^+/−^ plateau amplitude was smaller (36% decrease, *t*_(266)_ = 2.80, *p* = 0.038) compared to previously measured reduction in synaptic NMDAR currents (50%).

Despite their reduced amplitude, the duration of NMDAR plateau potentials was significantly prolonged in Y647S^+/−^ neurons, lasting over 200 ms beyond the WT neurons (Figure 2C). The area under the plateau potential for the 1 s period post-stimulation (late area) was significantly greater (181% increase) in Y647S^+/−^ neurons (Figure 2D; two-way ANOVA, genotype: F_(1, 127)_ = 36.74, *p* < 10^−4^; *t*_(127)_ = 5.33, *p* < 10^−4^, Sidak’s post hoc, *N* = 5, 7 mice for WT and Y647S). The total duration of plateau potentials was significantly increased in Y647S^+/−^ neurons (0.75 ± 0.09 s; Figure 2E) compared to WT (0.42 ± 0.03 s, *t*_(17)_ = 2.69, *p* = 0.015). NMDAR plateau potentials were blocked by the NMDAR antagonist D-AP5 (93% reduction) in both genotypes (two-way RM ANOVA, F_(1, 8)_ = 35.21, *p* < 10^−3^). The prolonged tail of Y647S^+/−^ plateau potentials was also sensitive to D-AP5 (72% reduction, *t*_(8)_ = 6.34, *p* = 0.0004, Sidak’s post hoc), indicating that it is an NMDAR-dependent phenomenon. Moreover, prolonged plateaus occurred in the absence of any major changes in intrinsic neuronal properties except for a small but significant decrease in capacitance and increase in excitability in *Grin1* Y647S^+/−^ mice (Table S1; Figure S1).

We considered whether differential expression of extrasynaptic NMDARs might contribute to prolonged plateau potentials, despite the observed reduction in isolated, synaptic NMDAR currents in Figure 1. Returning to those conditions that isolated NMDAR currents from other ion channels, we tested high frequency glutamate release, a protocol known to engage extrasynaptic receptors.^32^^,^^39^ Isolated NMDARs activated by this train showed 50% reduction in both amplitude (*t*_(16)_ = 2.197, *p* = 0.043) and duration in Y647S^+/−^ mice (Δ half-width = 120 ± 47 ms, *t*_(15)_ = 2.55, *p* = 0.022; Figure S2). This reduction in amplitude and faster kinetics, even at high-frequency stimulation, closely parallels the deficits in NMDAR EPSCs elicited by single pulse stimulation (Figure 1), suggesting that there is not selective enhancement of extrasynaptic responses. Instead, the data suggest that Y647S NMDARs in prefrontal cortex yield distinct electrophysiological responses depending on functional context (e.g., their typical ion channel milieu).

To further investigate the prolonged NMDAR plateau potentials in Y647S^+/−^ neurons, we performed simultaneous dendritic calcium imaging and whole-cell electrophysiology, as well as detailed morphological analysis of dendrites.

#### Dendritic Ca^2+^ influx initially reduced but has long tail in *Grin1* Y647S^+/−^ mice

**Figure 3:**
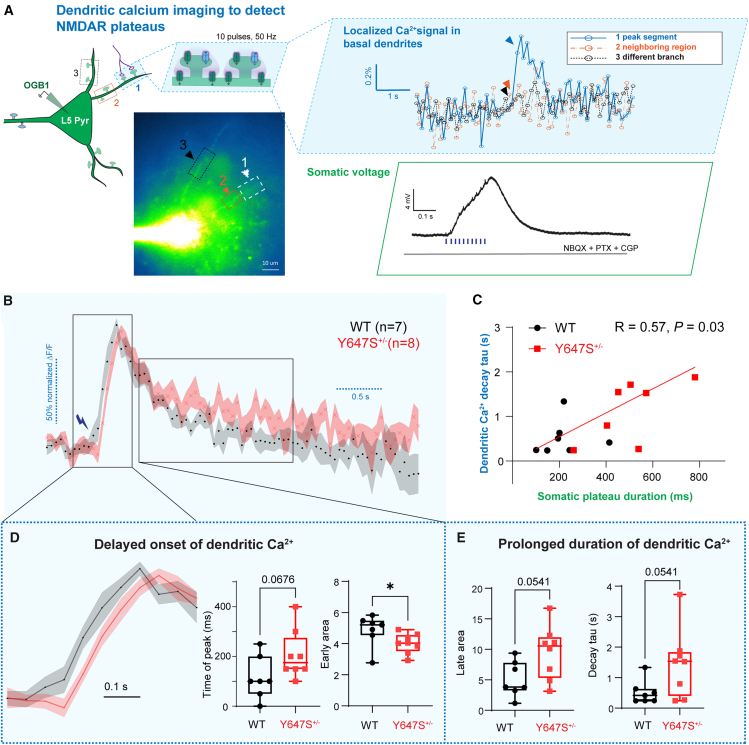
Delayed onset and prolonged duration of NMDAR-dependent dendritic Ca^2+^ influx in *Grin1* Y647S^+/−^ mice (A) Simultaneous somatic electrophysiology and dendritic Ca^2+^ imaging were performed with patch pipettes containing the fluorescent Ca^2+^ indicator Oregon Green BAPTA-1 (OGB1, 200 μM) and Alexa 594 (40 μM). Glutamate release onto basal dendrites was evoked by electrical stimulation (50 Hz, 10 pulses). Top right: ΔF/F in three dendritic segments, with localized NMDAR-dependent Ca^2+^ signal in the peak dendritic segment (1), but not in the neighboring region on the same branch (2), or a different branch (3). Bottom right: Somatic depolarization showing NMDAR-plateau potential in the same neuron. (B) Average normalized dendritic Ca^2+^ signal from WT (*n* = 7) and Y647S^+/−^ (*n* = 8) dendrites. (C) Decay time constant of dendritic Ca^2+^ signal is correlated with the duration of the somatic NMDAR plateau potential. (D) Early dendritic Ca^2+^ is reduced in Y647S^+/−^ neurons. Boxplots show delayed time of peak (left) and reduced area under response in the first 0.5 s (right) in Y647S^+/−^ dendrites. (E) Late dendritic Ca^2+^ shows prolonged elevation in Y647S^+/−^ neurons, with increased area under response (left) and increased decay time constant (right). ∗*p* < 0.05, Mann-Whitney test.

To directly assess local dendritic responses in *Grin1* Y647S^+/−^ neurons during NMDAR plateau potentials, we performed simultaneous patch-clamp electrophysiology and Ca^2+^ imaging in layer 5 pyramidal neurons under whole-cell recording conditions as in Figure 2. Using patch pipettes containing Oregon Green BAPTA-1 (OGB1), a fluorescent Ca^2+^ indicator, we detected highly localized NMDAR-mediated Ca^2+^ signals in basal dendrites during electrically evoked glutamate release (Figure 3A).

The pattern of changes in the dendritic Ca^2+^ spikes in Y647S^+/−^ dendrites mirror the genotype differences seen in the plateau potentials recorded at the cell body. The onset of dendritic Ca^2+^ influx in Y647S^+/−^ neurons is delayed compared to wild-type (WT) neurons (genotype Δ in time of peak = 91.96 ± 47.30 ms, 7 neurons from 3 WT mice, 8 neurons from 5 Y647S^+/−^ mice, Figures 3B–3E), and the area of the response immediately after stimulation (<0.5 s) is significantly reduced (*p* = 0.04). However, this initial deficit in Y647S^+/−^ dendritic Ca^2+^ response is followed by a prolonged elevation of dendritic Ca^2+^, with the 95% confidence intervals for Ca^2+^ decay time constant showing no overlap in WT (98–232 ms) and Y647S^+/−^ neurons (778–1386 ms). The prolonged dendritic Ca^2+^ elevation in Y647S^+/−^ neurons does not appear to be explained by the delayed onset, as the difference in decay time constant is 10-fold the difference in time of peak between WT and Y647S^+/−^ neurons. In contrast to the reduced initial Ca^2+^ response, the area under the later portion of the Ca^2+^ signal is also elevated in Y647S^+/−^ neurons (*p* = 0.0541), consistent with sustained dendritic excitation.

The duration of the NMDAR plateau potential recorded at the soma significantly correlates with the decay time of the dendritic Ca^2+^ signal (R = 0.57, *p* = 0.03; Figure 3C). These findings suggest that prolonged dendritic Ca^2+^ elevation contributes to altered excitability in *Grin1* Y647S^+/−^ mice.

#### Dendritic morphology is unaltered in *Grin1* Y647S^+/−^ mice

To probe whether dendritic properties are affected in *Grin1* Y647S^+/−^ mice, we investigated their dendritic morphology. Layer 5 pyramidal neurons were filled with neurobiotin during whole-cell patch clamp recordings, followed by post hoc fixation, immunostaining, and two-photon imaging.

We observed no significant differences in soma volume, apical or basal dendrite length, or dendritic branching as assessed by Scholl analysis (Figure S3). Additionally, spine densities in apical and basal dendrites were not different between the genotypes. These findings, along with the dendritic Ca^2+^ imaging results, support a causal link between altered NMDAR-dependent Ca^2+^ influx and prolonged dendritic integration in Y647S^+/−^ neurons.

Next, we investigated how the opposing loss and gain-of-function at different levels of NMDAR signaling affects cortical circuits in *Grin1* Y647S^+/−^ mice. We pursued this question using wide-field calcium imaging of cortical neuronal ensembles.

### Impact on cortical circuits

#### Extended neural population activity and epileptiform events in *Grin1* Y647S^+/−^ brain slices

**Figure 4:**
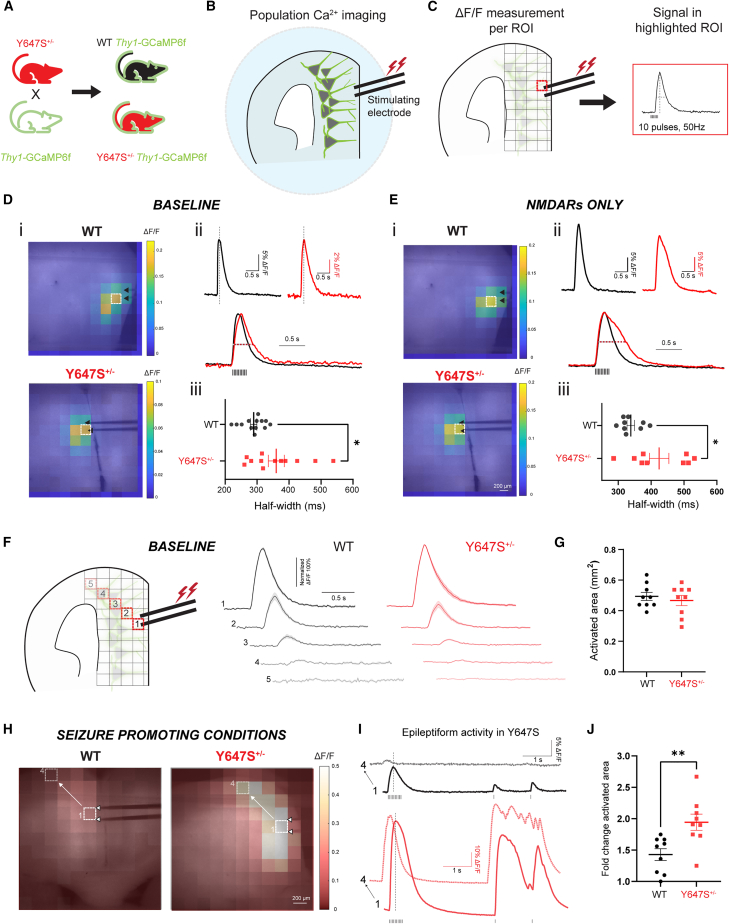
Prolonged neural population activity causes epileptiform events in *Grin1* Y647S^+/−^ brain slices (A) Schematic of breeding plan. Y647S^+/−^ mice were crossed with Thy1-GCaMP6f mice to generate WT and Y647S^+/−^ mice expressing GCaMP6f in layer 5 pyramidal neurons. (B, C) Wide-field calcium imaging of population neural activity in prefrontal brain slices. Stimulating electrode in the apical dendritic field of L5 neurons delivers 50Hz stimulation and the resulting fluorescence signal is captured on the widefield microscope. Change in fluorescence (ΔF/F) is calculated per 100-pixel square region (∼220 × 220 μm^2^). (D, E) (i) Heatmaps of ΔF/F at the peak signal superimposed on images of WT and Y647S^+/−^ brain slices under (D) baseline conditions (no pharmacological blockers) and (E) with only NMDARs active (AMPAR and GABARs blocked). Black arrows point out stimulating electrode position, white box indicates region with maximum signal. (ii) Fluorescence signal from region highlighted in (i) (top). Dotted line indicates time of peak signal shown in (i). Superimposed normalized responses in WT and Y647S^+/−^ are also shown (bottom). (iii) Comparison of signal half-width in WT and Y647S^+/−^ (∗*p* < 0.05, unpaired *t* test). Neural population activity is prolonged in Y647S^+/−^ brain slices without any blockers at baseline (D) and with only NMDARs active (E). (F) Spatial decay of population neural activity at baseline (no blockers) measured diagonally from the site of the stimulating electrode (left). Average fluorescence signal (normalized to the maximum) demonstrates similar spatial decay in WT and Y647S^+/−^. (G) Total activated area of slice in WT and Y647S^+/−^. (H) Heatmap of fluorescence signal under seizure promoting conditions in WT and Y647S^+/−^ (all GABARs blocked). Y647S^+/−^ slice shows larger activated area and greater signal. (I) WT slice (top) shows normal decay along the diagonal and very small responses to test stimuli delivered ∼4 s after the initial 50 Hz stimulation. (Bottom) Y647S^+/−^ slice exhibits reduced spatial decay and epileptiform activity with recurrent excitation triggered by the test stimuli. (J) Fold change in total activated area of the slice from baseline to seizure-promoting conditions in WT and Y647S^+/−^ (∗∗*p* < 0.01, unpaired *t* test).

To assess the circuit-level consequences of the Y647S NMDAR patient variant, we performed wide-field calcium imaging in brain slices from Y647S^+/−^ and littermate WT Thy1-GCaMP6f mice (Figures 4A–4C, *N* = 4 mice per genotype). The fluorescent calcium sensor GCaMP6f is well expressed in a large population of layer 5 excitatory neurons.^40^ We measured the temporal and spatial dynamics of cortical activity in response to electrically evoked glutamate release under specific pharmacological conditions.

The duration of population neural activity was significantly prolonged in Y647S^+/−^ mice compared to WT (Figure 4D; half-width, WT: 289 ± 10 ms, Y647S^+/−^: 360 ± 25 ms, Welch’s *t* test: *t*_(14.49)_ = 2.63, *p* = 0.019). Extended population activity in Y647S^+/−^ mice was observed even in the absence of any pharmacological blockers and with intact glutamatergic and GABAergic neurotransmission. Next, we pharmacologically isolated population NMDAR-mediated calcium signal using AMPAR and GABAR blockers (Figure 4E). Population NMDAR calcium signals were also significantly prolonged in Y647S^+/−^ brain slices (WT: 338 ± 11 ms, Y647S^+/−^: 425 ± 30 ms, *t*_(10.29)_ = 2.73, *p* = 0.02). We conclude that extended population neural activity is a direct circuit-level correlate of previously observed prolonged dendritic plateau potentials.

To determine whether spatial extent of cortical activity is altered in Y647S^+/−^ mice, we first measured the total activated area of the brain slice under baseline conditions with intact glutamatergic and GABAergic neurotransmission (Figure 4F). The total activated area of the slice was similar between WT and Y647S^+/−^ mice at baseline (Figure 4G; WT: 0.49 ± 0.03 mm^2^, Y647S^+/−^: 0.46 ± 0.03 mm^2^, *t*_(16)_ = 0.63, *p* = 0.53). However, under seizure-promoting conditions where GABAergic transmission was blocked, Y647S^+/−^ brain slices exhibited widespread epileptiform activity characterized by recurrent and persistent excitation even in the absence of stimulation (Figures 4H–4I). This resulted in larger area of the slice being activated in Y647S^+/−^ (Figure 4J; 1.9 ± 0.1-fold change from baseline) compared to WT (1.4 ± 0.1, *t*_(16)_ = 3.24, *p* = 0.005).

We conclude that the Y647S mutation results in prolonged cortical network excitation that can transition into widespread and persistent epileptiform activity. Our circuit-level characterization of a *GRIN1* patient variant mouse provides the first *ex vivo* proof of epileptiform activity and the conditions that can initiate seizures in mice and may do so in patients.

### Indirect manipulation of NMDAR kinetics reveals potential treatment strategy

Our investigation revealed deficient current through synaptic NMDARs, but paradoxically extended NMDAR integration and dendritic Ca^2+^ influx that result in cortical epileptiform activity in *Grin1* Y647S^+/−^ mice. Therefore, we targeted mechanisms that may indirectly prolong dendritic NMDAR excitation, despite the reduced amplitude and faster kinetics of receptor-level currents. The two mechanisms we considered are SK channel-mediated negative feedback, which curtails plateau potential duration, and magnesium block of NMDARs, which hastens channel closure.^41^^,^^42^ Under typical circumstances, normal Ca^2+^ influx through NMDARs activates small conductance SK calcium-activated potassium channels that hyperpolarize the membrane, promoting NMDAR Mg^2+^ block and thereby terminating further NMDAR activation.^33^^,^^41^^,^^42^^,^^43^ In Y647S^+/−^ neurons, reduced NMDAR-mediated Ca^2+^ influx could potentially result in insufficient activation of SK channels, causing prolonged dendritic plateau potentials.^44^ To identify opposing strategies and restore normal timing of dendritic excitation, we first targeted SK channel sensitivity in Y647S^+/−^ mice.

#### Boosting negative feedback: Potentiating SK channels prevents extended NMDAR integration in *Grin1* Y647S^+/−^

**Figure 5:**
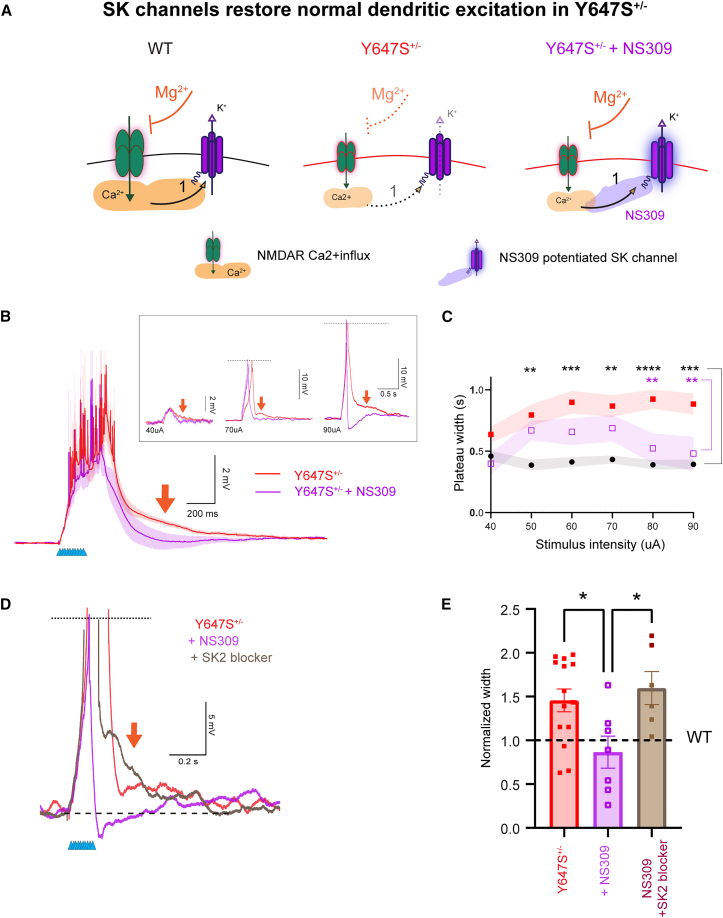
Potentiating SK channels restores normal NMDAR integration in *Grin1* Y647S^+/−^ mice (A) Schematic of potential mechanism showing impaired negative feedback in Y647S^+/−^ neurons due to insufficient Ca^2+^ influx compared to WT and the effect of NS309 in boosting SK channel Ca^2+^ sensitivity, thereby restoring negative feedback in Y647S^+/−^ neurons. (B) Average NMDAR plateau potential in Y647S^+/−^ neurons at 70 μA with extended tail indicated by red arrow. Application of 10 μM NS309 to the slice restores normal duration and terminates the NMDAR plateau potential in Y647S^+/−^ (Y647S^+/−^ + NS309). (Inset) Restoration of plateau potential duration by NS309 at increasing stimulus intensities in a Y647S^+/−^ neuron. (C) Total width of the NMDAR plateau potential in WT, Y647S^+/−^, and Y647S^+/−^+ NS309 neurons (∗∗*p* < 0.01, ∗∗∗*p* < 0.001, ∗∗∗∗*p* < 10^−4^, Tukey’s post hoc. Black stars: WT vs. Y647S^+/−^, purple stars: Y647S^+/−^ vs. Y647S^+/−^ + NS309). (D) SK2 channel blocker (Leidab7, 100 nM) prevents NS309 from reducing plateau potential duration in Y647S^+/−^ neurons. (E) Normalized NMDAR plateau width in Y647S^+/−^ neurons with the addition of NS309 and NS309+SK2 blockers (∗*p* < 0.05, Sidak’s post hoc).

We assessed whether the highly potent SK channel activator NS309 could reduce plateau potential duration in Y647S^+/−^ neurons to normal WT levels. NS309 increases the calcium sensitivity of SK channels,^45^^,^^46^ enabling their activation even with insufficient Ca^2+^ influx (Figure 5A). NS309 successfully eliminated the prolonged depolarization in Y647S^+/−^ neurons (Figure 5B; *N* = 4, 6 mice for WT and Y647S), restoring the duration of plateau potentials to WT levels (Figure 5C; plateau duration at highest stimulus: WT = 0.39 ± 0.04 s, Y647S^+/−^ = 0.88 ± 0.09 s, Y647S^+/−^ + NS309 = 0.48 ± 0.14 s).

NS309 can act on both SK2 and SK3 subtypes of SK channels in addition to intermediate conductance IK channels.^45^ To determine the channel subtype involved in NS309-restoration of plateau potential duration, we tested whether SK2 channels were the target by co-applying the SK2 blocker Leidab7. SK2 blockers occluded the effect of NS309, preventing the reduction in plateau potential duration in Y647S^+/−^ neurons (Figure 5D). Normalized plateau potential duration in Y647S^+/−^ (1.46 ± 0.13) was significantly reduced by NS309 (0.87 ± 0.18, *t*_(32)_ = 2.88, *p* = 0.028), but this decrease was prevented by the co-application of SK2 blockers (1.59 ± 0.19, *t*_(32)_ = 2.97, *p* = 0.022; Figure 5E). We conclude that the potentiation of SK2 channels by NS309 is sufficient to prevent prolonged dendritic excitation in Y647S^+/−^ neurons.

Next, we evaluated the impact of altering extracellular magnesium levels on Y647S^+/−^ neurons. This is of direct clinical relevance given the widespread prevalence of magnesium deficiency and its contribution to seizures in the general population.^47^

#### Reducing negative feedback: Lowering extracellular magnesium promotes NMDAR hyperexcitation in *Grin1* Y647S^+/−^

**Figure 6:**
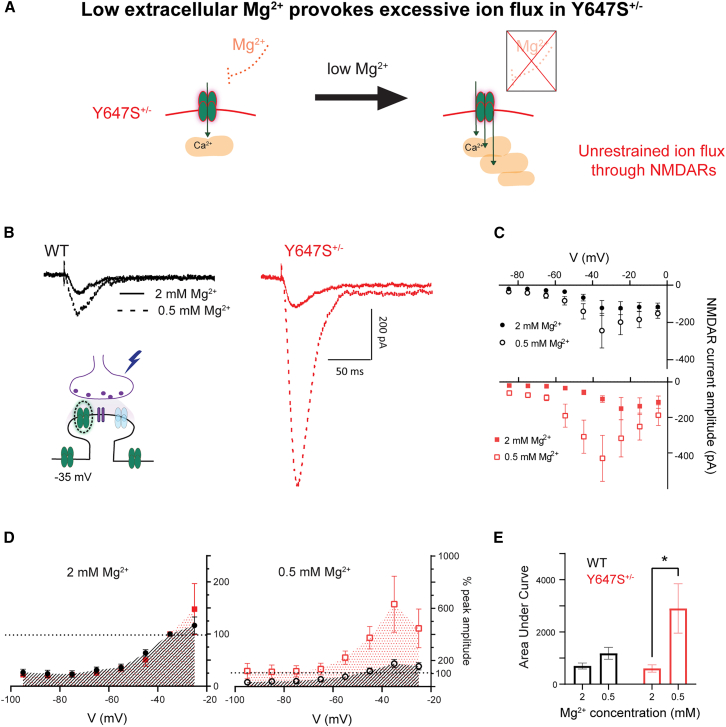
Reducing extracellular magnesium causes disproportionate NMDAR hyperexcitation in *Grin1* Y647S^+/−^ mice (A) Schematic illustrating the consequence of reducing extracellular Mg^2+^ levels which causes disproportionate current influx in Y647S^+/−^ neurons due to unrestrained NMDAR activation. (B) Electrically evoked NMDAR currents measured at holding potential of −35 mV in WT and Y647S^+/−^ neurons at 2 and 0.5 mM extracellular Mg^2+^ concentration. Potassium gluconate patch pipettes containing 5 mM QX314 were used to assess integrated NMDAR signaling with active dendritic ion channels. (C) Average current-voltage relationship (I-V curve) between NMDAR current amplitude and membrane potential at 2 and 0.5 mM Mg^2+^ in WT (top) and Y647S^+/−^ (bottom). (D) Normalized NMDAR activation curve (I-V curve normalized to peak at −35 mV, 2 mM Mg^2+^) in each genotype at 2 mM (left) and 0.5 mM (right) extracellular Mg^2+^ concentrations. (E) Area under the normalized NMDAR activation curve at 2 and 0.5 mM Mg^2+^ in WT and Y647S^+/−^ neurons (∗*p* < 0.05, Sidak’s post hoc).

We tested whether NMDARs activated by endogenous glutamate release in Y647S^+/−^ neurons would be disproportionately impacted by reduced extracellular Mg^2+^ (Figure 6A). To retain SK channel contribution, we used a modified patch solution with QX-314 and measured NMDAR currents at different membrane potentials (see “STAR Methods” under NMDAR EPSC IV curves). The shape of the current-voltage relationship (I-V) curve was similar in WT and Y647S^+/−^ mice at normal (2 mM) extracellular Mg^2+^, especially when comparing their area under curve (AUC: *t*_(26)_ = 0.14, *p* = 0.98). NMDAR current amplitude showed the expected increase with positive membrane potentials as Mg^2+^ block is relieved, reaching peak amplitude at −35 mV in both genotypes (Figure 6B). However, lowering extracellular Mg^2+^ from 2 to 0.5 mM caused a disproportionate increase in current amplitude only in Y647S^+/−^ neurons (6.3 ± 2.1-fold-change at −35 mV) compared to WT (1.8 ± 0.3-fold-change, *t*_(13)_ = 2.26, *p* = 0.04; Figures 6B and 6C). The overall area under the I-V curve is significantly increased in Y647S^+/−^ neurons at 0.5 mM Mg^2+^ (*t*_(26)_ = 2.61, *p* = 0.03; Figures 6D and 6E). These results show that Y647S^+/−^ neurons are specifically vulnerable to NMDAR hyperexcitation when extracellular Mg^2+^ is reduced.

Given the extreme vulnerability of Y647S^+/−^ neurons to decreases in extracellular Mg^2+^, we posited that increasing extracellular Mg^2+^ could be a viable treatment strategy to prevent seizures *in vivo*.

### Magnesium threonate treatment *in vivo* successfully treats seizures in *Grin1* Y647S^+/−^ mice

To boost NMDAR negative feedback *in vivo* and prevent seizures in Y647S^+/−^ mice, we aimed to increase brain levels of Mg^2+^. Magnesium supplementation has been historically used to treat seizures occurring during pre-eclampsia^48^^,^^49^ and other forms of epilepsy.^50^ However, previous formulations like magnesium sulfate do not achieve effective Mg^2+^ increase in the brain and require invasive administration.^51^ We opted for magnesium L-threonate, a recent formulation that can be administered orally to increase brain levels of Mg^2+^ with pro-cognitive effects.^35^^,^^52^^,^^53^ Adult littermate WT and Y647S^+/−^ mice of both sexes were given normal drinking water (control) or 0.5% w/v magnesium threonate (MgT) with *ad libitum* access for 12 weeks. We allowed for 6 weeks of pretreatment before assessing seizures to ensure efficacy well beyond the “honeymoon period” that is typical for antiepileptic drugs.^54^^,^^55^

**Figure 7:**
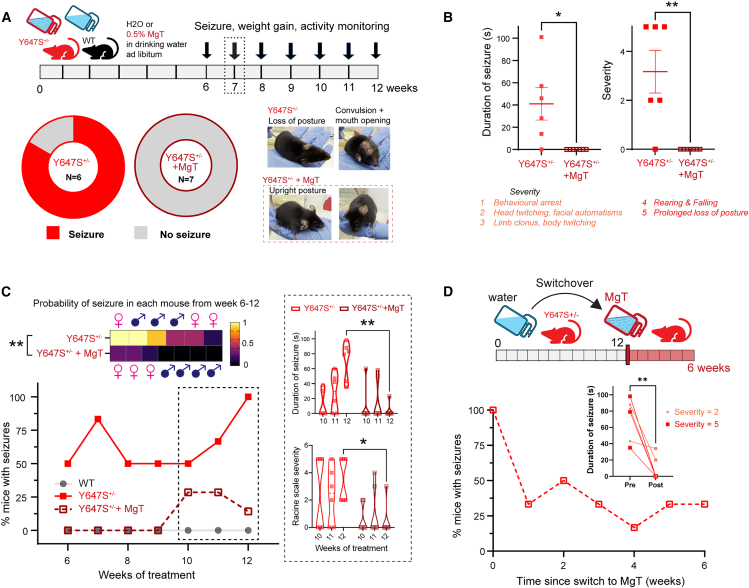
Magnesium threonate treatment *in vivo* successfully treats seizures in *Grin1* Y647S^+/−^ mice (A) Schematic of experimental plan. Adult WT and littermate Y647S^+/−^ mice were given *ad libitum* access to drinking water with 0.5% magnesium-L-threonate (MgT) or normal water (controls) for 12 weeks, with weekly monitoring of handling-induced seizures and weight gain from 6 weeks onwards. Donut charts show proportion of control Y647S^+/−^ (5/6) and Y647S^+/−^ + MgT (0/7) mice exhibiting handling-induced seizures at week 7 of treatment. Inset pictures show example convulsions with loss of posture and mouth opening in untreated Y647S^+/−^ mouse (top) versus normal upright posture in Y647S^+/−^ + MgT mouse. (B) Duration of seizures (left) and severity on our modified RS (right) in Y647S^+/−^ and Y647S^+/−^ + MgT mice at week 7 (∗*p* < 0.05, ∗∗*p* < 0.01, unpaired *t* test). (C) Seizure incidence and severity for the whole duration of the treatment. Top: Average seizure probability per mouse from 6 to 12 weeks shown as a heatmap, with sexes of individual mice shown above and below (∗∗*p* < 0.01, unpaired *t* test). Bottom: Percentage of mice with handling-induced seizures from weeks 6–12 of the treatment period. (Inset) Duration (top) and severity of seizures (bottom) in control and MgT-treated Y647S^+/−^ mice for weeks 10–12 (∗*p* < 0.05, ∗∗*p* < 0.01, Sidak’s post hoc). MgT remains effective in suppressing seizures until 12 weeks. (D) Top: Experimental schematic, untreated Y647S^+/−^ from the same cohort as C were switched to MgT treatment at 12 weeks. Bottom: Percentage of mice with seizures for 6 weeks from the switch. (Inset) Duration of seizures at week 0 (before switching to MgT) and at 6 weeks after switching to MgT (light red indicates mild seizures with severity 2, dark red corresponds to severe convulsive seizures, ∗∗*p* < 0.01, paired *t* test). Severe seizures are eliminated after switching to MgT.

At 7 weeks, 83% of untreated control Y647S^+/−^ mice (5/6) displayed prominent behavioral seizures upon handling, characterized by loss of posture, body and facial convulsions. Remarkably, none of the MgT treated Y647S^+/−^ mice (0/7) had seizures (Fisher’s exact test: *p* = 0.005; Figure 7A). Seizures in control Y647S^+/−^ mice lasted up to 1.7 min and were quite severe (mean: 41 ± 15 s, compared MgT treated Y647S^+/−^: *t*_(11)_ = 3.04, *p* = 0.011). These involved loss of posture, facial convulsions, limb clonus, and body twitching that were quantified by our modified Racine scale (RS), with 3/6 mice showing highest severity (RS 5). In contrast, MgT-treated Y647S^+/−^ mice did not display seizures of any severity (unpaired *t* test: *t*_(11)_ = 3.95, *p* = 0.002; Figure 7B). MgT appears to show 100% efficacy in preventing the spontaneous handling-induced behavioral seizures at 6–7 weeks of treatment. In addition, we observed MgT to be beneficial in improving weight gain and reducing hyperactivity in Y647S^+/−^ mice (Figure S4): weight gain in Y647S^+/−^ mice at 7 weeks of MgT treatment improved (67% of WT, *t*_(11)_ = 2.77, *p* = 0.02) compared to untreated Y647S^+/−^ mice (33% of WT). Running velocity was higher in Y647S^+/−^ mice compared to WT (*p* < 10^−4^) and reduced in MgT-treated Y647S^+/−^ mice (*p* < 10^−4^), showing no significant difference from WT (*p* = 0.47, Kruskal Wallis test).

We examined the long-term efficacy of MgT by monitoring the percentage of mice exhibiting seizures up to 12 weeks of treatment (Figure 7C). Seizures in control Y647S^+/−^ mice became more frequent, with all mice developing seizures by the 12^th^ week, and 2/6 mice having seizures every week. In contrast, MgT-treated mice showed no signs of seizures until 10 weeks, but a proportion (3/7) started to exhibit breakthrough seizures in weeks 10–12. The duration of seizures in weeks 10–12 was significantly reduced in MgT-treated Y647S^+/−^ mice compared to untreated control Y647S^+/−^ mice (two-way ANOVA, week x treatment: F_(2, 22)_ = 8.28, *p* = 0.002; Sidak’s post hoc: *t*_(5.98)_ = 6.16, *p* = 0.003). MgT-treated Y647S^+/−^ mice also had less severe seizures compared to untreated controls (two-way ANOVA, treatment: F_(1, 11)_ = 5.763, *p* = 0.03; Sidak’s post hoc at week 12: *t*_(33)_ = 3.11, *p* = 0.011). Breakthrough seizures in MgT-treated mice all occurred in female mice (*N* = 3/7; Figure 7C) indicating that females might be more vulnerable to reductions in treatment efficacy. Overall, the average seizure probability was significantly higher in control Y647S^+/−^ mice (0.59 ± 0.14) compared to MgT-treated Y647S^+/−^ mice (0.11 ± 0.05, unpaired *t* test: *t*_(11)_ = 3.36, *p* = 0.034).

Finally, we examined whether acute treatment with MgT could be effective in the untreated Y647S^+/−^ mice that had developed severe seizures (Figure 7D). This experiment was critical to determine whether treatment in adulthood after full development of disease pathology could still be effective. Control Y647S^+/−^ mice that were switched to MgT treatment at week 12 showed an immediate reduction in seizure occurrence with complete elimination of severe seizures after 2 weeks of treatment. The duration of seizures was significantly reduced in all mice after 6 weeks of MgT treatment (paired *t* test: *t*_(5)_ = 4.54, *p* = 0.006). We conclude that MgT intervention is acutely effective in reducing seizure occurrence and severity in *Grin1* Y647S^+/−^ mice.

Our work reveals an intriguing condition where loss-of-function GluN1 variant NMDARs paradoxically prolong cortical excitation and promote seizures. We show that enhancing NMDAR negative feedback with oral magnesium successfully mitigates seizures despite substantial receptor dysfunction, revealing a promising treatment strategy.

## Discussion

Our work in *Grin1* Y647S^+/−^ mice reveals opposing consequences of a human GluN1 variant on synaptic, dendritic, and circuit-wide NMDAR signaling. Despite a major loss of NMDAR function, dendritic NMDAR signaling is excessively prolonged, promoting seizures. These substantial neurophysiological deficits occur without changes in neuronal morphology. We show that boosting negative feedback of NMDARs restores normal excitation kinetics and successfully treats seizures *in vivo*.

### Reconciling loss/gain of function of NMDAR patient variants with multi-scale assessment

The GluN1 Y647S variant is predicted to be loss-of-function^8^^,^^21^^,^^56^ based on decreased currents and surface expression in oocytes,^21^ HEK cells, and neurons.^23^ But there are also reports of increased agonist affinity in Y647S and Y647C GluN1 variant NMDARs.^8^^,^^57^ Previous work has shown that severe knockdown of the GluN1 subunit has subcellular compartment-specific consequences,^32^ prompting our comprehensive assessment in prefrontal neurons in *Grin1* Y647S^+/−^ mice. We found reduced current through synaptic NMDARs, consistent with receptor loss-of-function. However, dendritic and circuit-level NMDAR signaling was greatly enhanced, promoting seizure vulnerability. These opposing effects show that functional context dictates NMDAR deficits and receptor-level impact is insufficient to characterize patient variants.

The Y647S variant has a complex phenotype that is not a simple consequence of reduced protein expression. It is likely shaped by lifelong compensatory processes involving dendritic ion channels triggered by unexpected NMDAR channel properties. A concatenation of compensatory processes may make it difficult to distinguish the immediate cause: altered NMDAR channel properties and surface expression, from the end disease pathology: circuit wide hyperexcitation that results in seizures and cognitive dysfunction.

There is growing recognition that integrative parameters are necessary to understand glutamate receptor patient variants.^14^^,^^58^^,^^59^^,^^60^ Yet, there is a bias toward receptor-level impact when classifying ion channel variants, especially in emerging machine learning techniques.^61^^,^^62^ While receptor-level impact is a first step to predict variant pathogenicity, functional context is essential to determine overall phenotype and predict treatments.

### Dendritic plateau potential kinetics provide crucial insight into NMDAR variant pathology

Plateau potentials occur in thin dendrites of pyramidal neurons when extrasynaptic NMDARs are activated by glutamate spillover.^38^^,^^63^^,^^64^ Plateau potentials are linked to cognitive integration,^65^ synaptic plasticity,^29^ and enable the formation of neuronal ensembles.^64^^,^^66^ We identified a small but significant reduction in plateau potential amplitude along with severely prolonged duration in *Grin1* Y647S^+/−^ neurons. Consistently, dendritic calcium imaging revealed reduced initial Ca^2+^ influx, followed by a prolonged tail in Y647S^+/−^ neurons. This prolonged tail is NMDAR-dependent but may include contributions from other sources such as voltage-gated calcium channels.

Plateau potential properties are closely linked to dendritic calcium-activated potassium channels (SK and BK)^44^^,^^67^ and are known to alter excitability in Fragile X^68^^,^^69^ and Dravet syndrome.^70^ NMDAR Ca^2+^ influx activates potassium channels that provide essential negative feedback by helping to restore NMDAR magnesium block.^71^^,^^72^ High-affinity SK channels on dendritic shafts and synapses exert powerful inhibition of NMDAR postsynaptic potentials.^33^^,^^44^^,^^73^^,^^74^ We found that enhancing calcium sensitivity of SK channels in Y647S^+/−^ mice restored normal plateau potential duration. In contrast, lowering extracellular Mg^2+^ levels unmasked the propensity of Y647S neurons to trigger NMDAR hyperexcitation. Despite having intact Mg^2+^ block under physiological conditions,^25^^,^^57^ Y647S^+/−^ NMDARs were highly sensitive to small decreases in external magnesium. We interpret this as a failure of mutant receptors in engaging negative feedback mechanisms that typically restrain NMDAR excitation (graphical summary).

### Magnesium supplementation to treat seizures caused by GRIN1 variant

Treatments for *GRIN* disorder have previously focused on direct receptor agonism/antagonism. NMDAR antagonists ketamine and memantine reduced seizure burden in a GluN1 gain-of-function patient,^9^ and glutamate receptor antagonists radiprodil^75^^,^^76^ and perampanel^77^^,^^78^ are effective for other gain-of-function variants. L-serine, a precursor of NMDAR co-agonist D-serine, improves motor and cognitive functions with limited effect on seizures in loss-of-function variants.^79^^,^^80^^,^^81^ However, seizures in loss-of-function GluN1 variants may arise from compensatory processes downstream of the receptor due to its obligatory nature. We show that despite Y647S receptors being loss-of-function, indirect effects result in protracted excitation and seizure vulnerability that are sensitive to external magnesium changes. Therefore, we chose to suppress seizures using magnesium supplementation.

Magnesium sulfate treatment is well-supported for seizures in preeclampsia^48^ and other forms of epilepsy.^50^^,^^82^ However, it has been difficult to increase brain levels of Mg^2+^ efficiently with previous Mg^2+^ formulations and required invasive administration intrathecally.^51^ Furthermore, assessing spontaneous seizures in *Grin1* Y647S^+/−^ mice poses challenges that shaped our treatment approach. Acute drug administration via injections or intracortical infusion requires extensive handling and anesthetic exposure, which can alter NMDAR activity. Additionally, handling the mice during injections may itself trigger seizures, reducing the likelihood of observing spontaneous seizures in the following hours and complicating treatment efficacy assessment. Magnesium threonate (MgT) is well transported to the brain via oral administration,^35^ supports brain health and cognition,^83^^,^^84^ making it a superior alternative.

We implemented a minimally invasive treatment with *ad libitum* MgT in the drinking water, coupled with weekly handling to monitor spontaneous seizures. Remarkably, MgT treatment substantially reduced the incidence and severity of seizures in *Grin1* Y647S^+/−^ mice chronically over 3 months, and acutely within 2 weeks. The average western diet is deficient in magnesium^85^ and severe hypomagnesemia causes seizures.^86^ Genetic variants in magnesium transporters are also linked to risk of several neuropsychiatric illnesses.^87^ It is critical to evaluate and treat *GRIN* disorders against the backdrop of common dietary and genetic vulnerability in brain magnesium levels.

### Significance

Using mice with a patient-variant in the obligate GluN1 NMDAR subunit, we show that seizure-promoting excitation arises from loss-of-function via impaired negative feedback. We successfully target this unexpected mechanism to treat seizures, demonstrating the importance of functional context to decipher and treat NMDAR dysfunction in *GRIN* disorder.

### Limitations of the study

We limited our examination to principal neurons of the cortex and explored only one potential mechanism involving SK channels that influences aberrant dendritic excitatory kinetics in *Grin1* Y647^+/−^ mice. Further examination may shed light on other ion channels or extrasynaptic NMDARs that shape aberrant dendritic excitation in these mice. Measuring currents evoked by exogenous application of the agonist NMDA may further provide an independent and complementary measure of NMDAR activity. In addition, interneuron function should be investigated in this model, since there are reports that NMDAR antagonists reduce interneuron firing and lead to cortical disinhibition.^88^^,^^89^^,^^90^

To suppress seizures, we used oral magnesium-L-threonate supplementation which was highly effective. While we do not explicitly confirm that MgT increased brain Mg^2+^ levels in our mice, the effect of magnesium supplementation on CSF Mg levels are well-documented.^35^^,^^91^^,^^92^ In addition to Mg^2+^ supplementation, testing the efficacy of NS309 injections in suppressing seizures would be an exciting experiment to pursue in future work. While we focused on reducing seizures in *Grin1* Y647S^+/−^ mice, the core deficit remains a substantial loss of NMDAR function which may impact cognitively critical phenomena such as synaptic plasticity.^25^ These effects are not easily separable from the detrimental cognitive burden and long-term effects of severe seizures. An effective treatment for seizures represents an essential step forward, benefiting future assessment of other neurological changes in *GRIN* disorder.

## Resource availability

### Lead contact

Requests for further information and resources should be directed to and will be fulfilled by the lead contact, Evelyn K. Lambe (evelyn.lambe@utoronto.ca).

### Materials availability

This study did not generate new unique reagents.

### Data and code availability

• All data reported in this paper are available from the lead contact upon request.
• This paper does not report original code.
• Any additional information required to reanalyze the data reported in this paper is available from the lead contact upon request.

## Acknowledgments

We thank Wendy Horsfall for expert technical assistance. This research was generously funded by 10.13039/501100000024CIHR (PJT-178372, EKL), 10.13039/100014370SFARI (AJR), Cure GRIN (AJR), Ontario Graduate Scholarships (SV), and Schmidt Science Fellowship (SV).

## Author contributions

Conceptualization, S.V. and E.K.L.; investigation, S.V., D.N., M.T.S., Y.-F.T., and S.Q.; writing – original draft, S.V. and E.K.L.; writing – review and editing, S.V., D.N., M.T.S., Y.-F.T., S.Q., A.J.R., and E.K.L.; visualization, S.V. and E.K.L.; funding acquisition, S.V., A.J.R., and E.K.L.; resources, A.J.R. and E.K.L.; supervision, S.V., A.J.R., and E.K.L.

## Declaration of interests

As a member of the scientific advisory board of the CureGRIN Foundation, A.J.R. has received financial remuneration.

## STAR★Methods

### Key resources table

**Table:** 

REAGENT or RESOURCE	SOURCE	IDENTIFIER
**Chemicals, peptides, and recombinant proteins**		

NBQX	Alomone Labs	*N*-186
CNQX	Alomone Labs	C-141
D-AP5	Alomone Labs	D-145
TTX	Alomone Labs	T-550
Picrotoxin	Alomone Labs	P-325
CGP-52432	Tocris	1246
NS309	Tocris	3895
Cesium gluconate	Hellobio	HB4822
QX-314 bromide	Alomone	Q-100
Magnesium L Threonate	AK Scientific	W8428

**Experimental models: Organisms/strains**		

Grin1 Y647S ^±^	Gift from Dr. Amy Ramsey at University of Toronto	NA
Thy1-GCaMP6f	Jackson Laboratory	RRID:IMSR_JAX:025393

**Software and algorithms**		

Clampfit 10.7	Molecular Devices	NA
AxoGraph X	AxoGraph	NA
MATLAB R2023a	Mathworks	NA
Neurolucida 360	Mbf Bioscience	NA

### Experimental model details

Mice heterozygous for the Y647S mutation in the *Grin1* gene encoding for the obligate GluN1 subunit (*Grin1* Y647S^+/−^ mice) were generated as described previously.^25^ Thy1-GCaMP6f mice (RRID: IMSR_JAX:02828) were crossed with *Grin1* Y647S^+/−^ mice and the offspring which were positive for Thy1-GCaMP6f and Y647S^+/−^ or WT were used for widefield calcium imaging and subsequent *in vivo* treatments. Adult (>P75 days) mice on a C57Bl/6J background were used for all experiments, with both male and female mice included in equal proportions when possible and balanced across genotypes. Major comparisons were analyzed for sex differences and interactions, and none were detected. Age was balanced across genotypes. In the electrophysiological experiments, the mice were 155 ± 12 days, and those that were treated and followed for the behavioral studies were 84 ± 4 days at the start of the experiment. All experiments were approved by the Temerty Faculty of Medicine Animal Care and Use Committee (protocol# 20011621) and followed Canadian Council on Animal Care and AVMA guidelines. Mice were group-housed and kept on a 12-h light cycle with food and water access *ad libitum*.

### Method details

#### Brain slicing

Prefrontal cortex coronal brain slices (400 μm) were extracted in ice-cold sucrose ACSF (in mM: 254 sucrose, 10 days-glucose, 26 NaHCO_3_, 2 CaCl_2_, 2 MgSO_4_, 3 KCl and 1.25 NaH_2_PO_4_) and allowed to recover for ∼2 h in oxygenated (95% O_2_, 5% CO_2_) ACSF (in mM: 128 NaCl, 10 D-glucose, 26 NaHCO_3_, 2 CaCl_2_, 2 MgSO_4_, 3 KCl, and 1.25 NaH_2_PO_4_) at 30°C. For whole-cell electrophysiology or wide-field calcium imaging, brain slices were transferred to the stage of a BX51WI microscope (Olympus, Tokyo, Japan) and perfused with oxygenated ACSF at 30°C. Recording electrodes (2–4 MΩ) filled with patch solution were used to record from layer 5 (L5) pyramidal neurons. Multiclamp 700B amplifier with Digidata 1440A and pClamp 10.7 software (Molecular devices) were used for data acquisition at 20 kHz. All recordings were compensated for liquid junction potential.

#### Whole-cell electrophysiology

##### Patch solutions

*Regular patch solution (K-gluconate):* in mM, 120 potassium gluconate, 5 KCl, 10 HEPES, 2 MgCl_2_, 4 K_2_-ATP, 0.4 Na_2_-GTP, and 10 sodium phosphocreatine, pH 7.3. Unless otherwise specified, K-gluconate patch solution was used for most experiments, with the sodium channel blocker QX-314 (5 mM) added in a subset of specified experiments.

*Cesium gluconate patch solution (Cs-gluconate):* in mM, 105 Cesium gluconate, 17.5 CsCl, 10 HEPES, 10 BAPTA Tetracesium salt, 2 Mg-ATP, 0.3 Na_2_-GTP, 5 QX-314 bromide, pH adjusted to 7.3 with CsOH.

##### Protocols for NMDAR assessment

###### Evoked EPSCs

Pharmacologically isolated α-amino-3-hydroxy-5-methyl-4-isoxazolepropionic acid receptor (AMPAR) and NMDAR-mediated evoked excitatory postsynaptic currents (EPSCs) were measured using Cs-gluconate patch pipettes in voltage-clamp at a holding potential of −60 and +40 mV respectively. Cs-gluconate pipettes allowed us to isolate currents through the glutamate receptors by blocking all other sodium, potassium and calcium ion channels that might get activated at more positive membrane potentials. A bipolar stimulating electrode (FHC) in the basal dendritic field ∼100 μm from the soma of the recorded layer 5 pyramidal neuron was used to stimulate synaptic glutamate release with single pulses of 40 μs duration delivered at 0.1 Hz. To calculate synaptic AMPA/NMDA ratios, the NMDAR component of the EPSC at +40 mV was taken to be the amplitude at 3 ∗ tau_decay_ of the AMPAR EPSC at −60mV. NMDAR EPSC half-width was measured after normalizing the amplitudes of all cells. Gamma-aminobutyric acid receptor (GABAR) blockers (Picrotoxin 20 μM, *Alomone Labs*, CGP52432 1 μM, *Tocris*) were included in all recordings, and AMPAR blocker (CNQX or NBQX 20 μM, *Alomone Labs*) was used to isolate synaptic NMDAR currents at +40 mV.

Miniature EPSCs (mEPSC) were measured in the presence of sodium channel antagonist tetrodotoxin (TTX, 2 μM) to examine presynaptic release probability and postsynaptic strength.

###### NMDAR plateau potentials

To measure the impact of the Y647S mutation on NMDAR-dependent dendritic integration, we evoked basal dendritic plateau potentials,^28^^,^^32^^,^^93^ by delivering 10 pulses of 50 Hz stimuli in the presence of AMPAR and GABAR blockers (Picrotoxin 20 μM, CGP52432 1 μM, NBQX 20 μM). Current clamp recordings at −75 mV were obtained using K-gluconate patch solution. Amplitude and duration of NMDAR plateau potentials was measured using Axograph and Clampfit 10.2 (Molecular Devices). The peak amplitude of NMDAR plateau potentials was capped at the spiking threshold, and peaks were normalized to the average spike threshold for comparison across stimulus intensities and genotypes. The total width of the plateau potential, and area under the plateau potential for 1s post-stimulation (late area) were used to assess the duration of the plateau potentials. D-AP5, 50 μM was used to confirm NMDAR dependence of plateau potentials. SK channel positive allosteric modulator NS309 (10 μM, *Tocris*) was included in a subset of experiments to restore appropriate duration of dendritic integration in Y647S^+/−^ mice. SK2 channel blocker Leidab7 (100 nM) was included in a subset of experiments along with NS309 to determine the contribution of SK2 channels to NS309’s effect.

###### NMDAR EPSC IV curves

K- gluconate patch pipettes with 5 mM QX-314 were used to measure NMDAR EPSCs at positive membrane potentials while also keeping other ion channel contributions intact. Mainly this allowed us to assess SK channel mediated negative feedback of NMDARs. I-V curves were generated by recording NMDAR-EPSCs in response to basal dendritic electrical stimulation in the presence of AMPAR and GABAR blockers at different membrane potentials (−85 to −5 mV).

#### Dendritic calcium imaging

To simultaneously record dendritic calcium signals and electrophysiological responses, we filled layer 5 pyramidal neurons with the fluorescent Ca^2+^ indicator Oregon Green BAPTA-1 (OGB-1, 200 μM) and Alexa 594 (40 μM) to visualize dendrites. Fluorescent dendritic Ca^2+^ signals were measured at 50 ms exposure in response to electrically evoked glutamate release. Kinetics of the calcium signal were compared after normalizing the peak ΔF/F across all responses per genotype.

#### Neuronal morphology

To quantify neuronal morphology, neurons were filled with 0.5% Neurobiotin during whole-cell electrophysiology. Slices were then fixed and immunostained using streptavidin conjugated Alexa 594, followed by two photon imaging (*Bruker*), morphology tracing, and analysis (*Neurolucida 360)*.

#### Widefield calcium imaging

To characterize the circuit-wide effect of the *Grin1* Y647S mutation, an optical measure of population level neuronal activity was recorded from WT and Y647S^+/−^mice expressing GCaMP6f under the *Thy1* promoter (*Thy1*-GCaMP6f) in layer 5 pyramidal neurons. Population neural activity in cortical brain slices was evoked by apical dendritic stimulation (10 pulses, 50 Hz) in layer 2/3 and measured using the Prime BSI Express camera (*Teledyne*) at 20 ms exposure through the 10X lens of the widefield microscope.

Image acquisition was binned every 2 pixels, and the resulting 1024 x 1024-pixel image was subdivided into square regions of 100 x 100 pixels (∼220 × 220 μm^2^). Fluorescence intensity change (ΔF/F) over baseline in each subregion of the slice was quantified, and kinetics and spatial spread of population activity were measured. ImageJ and MATLAB were used for analysis. Since different slices have varying strengths of GCaMP6f expression, we normalized all responses to the highest response within that slice and compared the kinetics of peak-normalized calcium signals across slices. Half-width (ms) of calcium signals was determined at the region with the highest ΔF/F response. The area under the ΔF/F curve (AUC) for 2s following the electrical stimulus was determined for all regions in the slice and the total number of regions with AUC >1 Standard Deviation from the mean was taken as the total activated area of the slice. GABAergic blockers (Picrotoxin 20 μM, CGP52432 1 μM) were used to mimic seizure-promoting conditions. To examine persistent activity, two single pulse stimuli were delivered 1.5 s apart ∼4 s following the initial 10 pulse stimulation. Epileptiform activity in Y647S^+/−^ mice was characterized by a large amplitude response to the single pulse stimulus that persisted continuously with recurrent excitation even in the absence of any stimulation in the intervening period.

#### Magnesium threonate treatment and seizure characterization

*Grin1* Y647S^+/−^ and WT littermate animals (84 ± 4 days old) were given either 0.5% w/v Magnesium L-Threonate (*AK scientific*) dissolved in their drinking water (MgT) or regular tap water (Control). Mice had access to water *ad libitum* and the MgT dose was calculated to be 1086 mg/kg (for 23g mice drinking 5 mL per day). This equates to an elemental magnesium dose of 88.5 mg/kg per day. Mice were picked up and handled by the experimenter once a week at the same time of day (11 a.m.) for ∼2–3 min, followed by weighing the mice. Seizures occurring during this period were quantitatively assessed from video recordings of Y647S^+/−^ mice from both MgT and control groups by an independent observer. Mice underwent cage changes and water bottle switches on the day following the handling induced seizure assessment. *Grin1* Y647S^+/−^ mice reliably seize upon handling once every week, but more frequent handling or cage manipulation reduces the frequency of seizures. Therefore, we chose a minimally invasive treatment protocol with *ad libitum* oral administration of MgT and weekly handling.

After the initial 12 weeks experiment, control Y647S^+/−^ mice that displayed prominent seizures on vehicle were switched to MgT treatment and their seizure incidence was monitored for an additional 6 weeks. Videos containing visually identifiable seizures were assessed from weeks 6–12 of the treatment period. In total, 50 videos were analyzed and total seizure duration in seconds, exhibited symptoms, and seizure severity were measured as per our modified Racine scale^94^^,^^95^ (0 - normal behavior, 1 - behavioral arrest and vacant stare, 2 - head nodding, facial automatisms, tail extension, 3 - unilateral clonus, 4 - bilateral clonus, rearing and falling, 5 - loss of posture). All animals were humanely euthanized after the experiment and re-genotyped.

#### Open field behavior testing and tracking

On the 7^th^ week of MgT treatment, mice were handled for ∼2 min for seizure assessment and then placed in a circular open field arena where they were allowed to explore for 10 min under continuous overhead camera monitoring. The arena was cleaned thoroughly between each mouse. DeepLabCut^96^ and Bonsai^97^ were used to track animal body parts. Total distance traveled and velocity of the mice were calculated using DLCAnalyzer.^98^

### Quantification and statistical analysis

Statistical tests were performed in Prism 9 (Graphpad). Data are presented as mean ± SEM. Comparisons of electrophysiological properties between WT and Y647S^+/−^ mice used two-tailed unpaired t-tests or Welch’s test if the variance was different between the groups. two-way ANOVA or two-way repeated measure (RM) ANOVA with Sidak’s post hoc test were used when considering the interaction between genotype and other variables such as stimulus intensity or pharmacological blockers. Within-cell effects of pharmacological manipulations were evaluated using paired t-tests. One-way ANOVA with Tukey’s post hoc test was used to evaluate efficacy of treatments in restoring Y647S^+/−^ properties to WT levels. Distribution of running velocity across groups of mice was compared using Kruskal Wallis test. ∗*p* < 0.05, ∗∗*p* < 10^−2^, ∗∗∗*p* < 10^−3^, ∗∗∗*p* < 10^−4^
