# Supplementary material for: Context-dependent NMDA receptor dysfunction predicts seizure treatment in mice with human GluN1 variant
Source: iScience. 2025 Dec 1;29(1):114301. doi: 10.1016/j.isci.2025.114301 (PMC12834114; doi:10.1016/j.isci.2025.114301)
Supplement: Document S1. Table S1 and Figures S1–S4 [file mmc1.pdf]

iScience, Volume *29*

Supplemental information

Context-dependent NMDA receptor dysfunction predicts seizure treatment in mice with human GluN1 variant

Sridevi Venkatesan, Daria Nazarkina, Megan T. Sullivan, Yao-Fang Tan, Sarah Qu, Amy J. Ramsey, and Evelyn K. Lambe

Supplemental Material (Venkatesan et al)

Supplemental Table S1. Intrinsic electrophysiological properties of layer 5 pyramidal neurons. Supplementary Figure S1

Supplemental Figure S1. Intrinsic excitability is slightly increased in *Grin1* Y647S$^{+/-}$ neurons

Supplemental Figure S2. Reduced amplitude and faster kinetics of NMDAR currents even at high frequency stimulation in *Grin1* Y647S$^{+/-}$ mice

Supplemental Figure S3. Morphological properties of layer 5 pyramidal neurons are unaltered in Grin1 Y647S$^{+/-}$ mice

Supplemental Fig S4. Magnesium threonate improves weight gain and restores normal running velocity in *Grin1* Y647S$^{+/-}$ mice at 7 weeks of treatment

Supplemental Table S1

	WT (n = 16)	Y647S$^{+/-}$ (n = 19)	T-test statistics
Resting membrane potential (mV)	-83 ± 2	-84 ± 1	$t_{(33)} = 0.52$, $P = 0.61$
Input resistance (MΩ)	124 ± 4	119 ± 9	$t_{(33)} = 0.36$, $P = 0.76$
Membrane Capacitance (pF)	112 ± 4	95 ± 6	$t_{(33)} = 2.27$, $*P = 0.03$
Action potential threshold (mV)	-48 ± 1	-50 ± 1	$t_{(33)} = 1.23$, $P = 0.23$
Rheobase (pA)	122 ± 13	110 ± 10	$t_{(31)} = 0.73$, $P = 0.47$

Supplementary Table 1. Intrinsic electrophysiological properties of layer 5 pyramidal neurons. Layer 5 pyramidal neurons in the medial prefrontal cortex of WT and Y647S$^{+/-}$ mice show similar intrinsic electrophysiological properties, except for membrane capacitance, which is significantly decreased in Y647S$^{+/-}$ neurons. **(related to Fig 2)**

Supplemental Figure S1

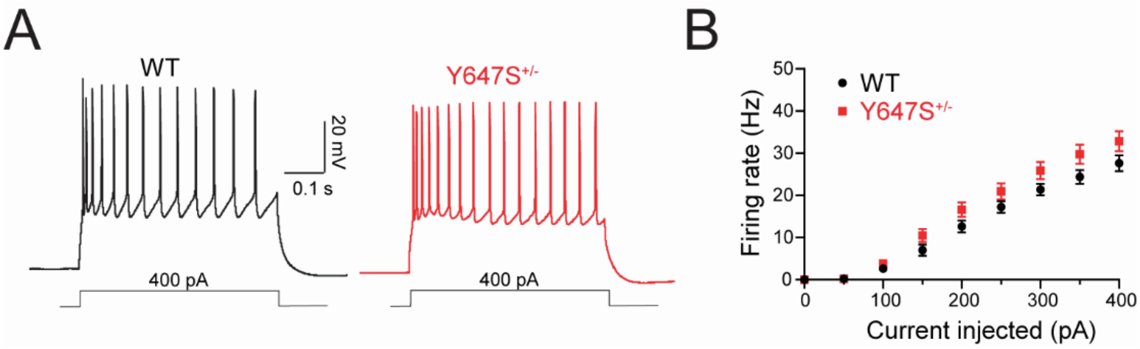

Supp Fig S1. Intrinsic excitability is slightly increased in *Grin1* Y647S$^{+/-}$ neurons. A, Action potentials in layer 5 pyramidal neurons with 400 pA constant current injection for 0.5 s in WT and *Grin1* Y647S$^{+/-}$. **B,** Input output curve showing action potential frequency at different current injections. Difference in slope of input-output curve: $F_{(1, 311)} = 6.68$, $P = 0.009$. There is no significant difference in firing rate at any individual current injection (Sidak's post hoc after 2-way ANOVA). **(related to Fig 2)**

Supplemental Figure S2

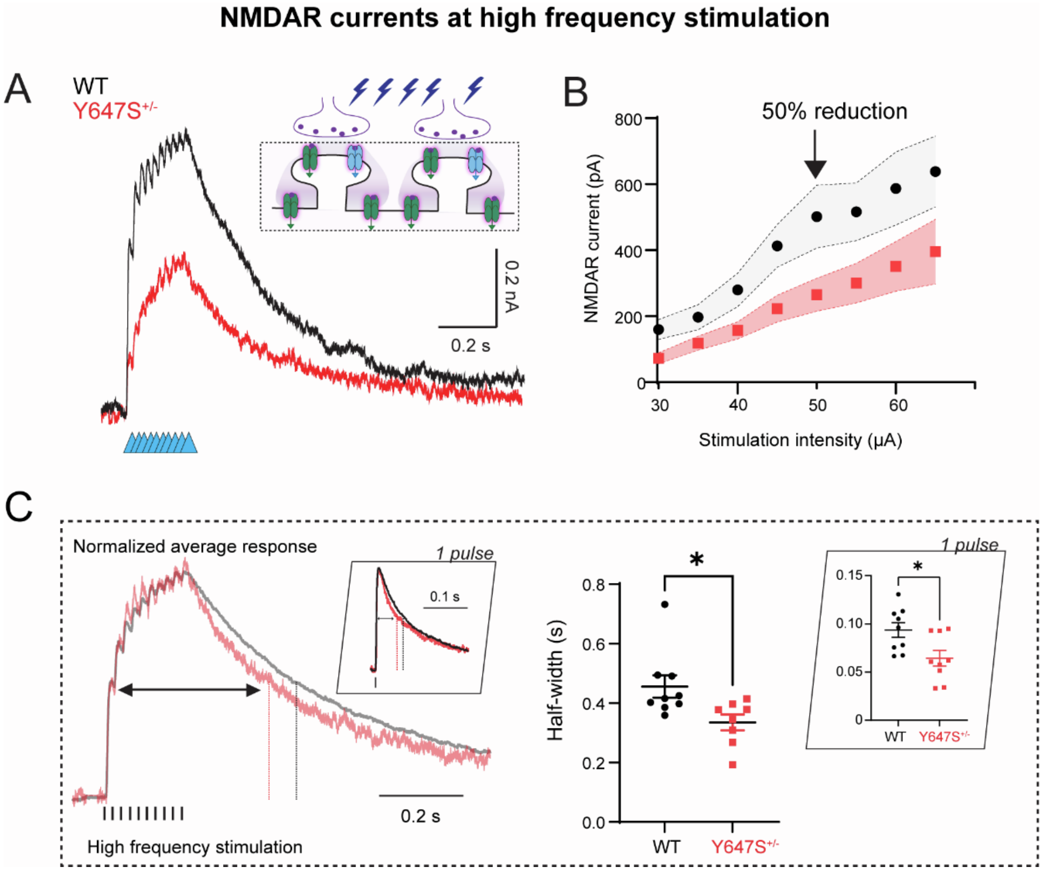

Supp Fig S2. Reduced amplitude and faster kinetics of NMDAR currents even at high frequency stimulation in *Grin1* Y647S$^{+/-}$ mice

A, NMDAR currents measured with cesium gluconate internal solution in response to high frequency stimulation (10 pulses, 50 Hz). **B,** Peak amplitude of NMDAR currents is significantly reduced in Y647S$^{+/-}$ neurons even at high frequency stimulation. **C,** Average peak normalized NMDAR currents reveal significantly faster decay in Y647S$^{+/-}$ neurons both at high frequency and single pulse stimulation (*$P < 0.05$, unpaired t-test). **(related to Fig 2)**

Supplemental Figure S3

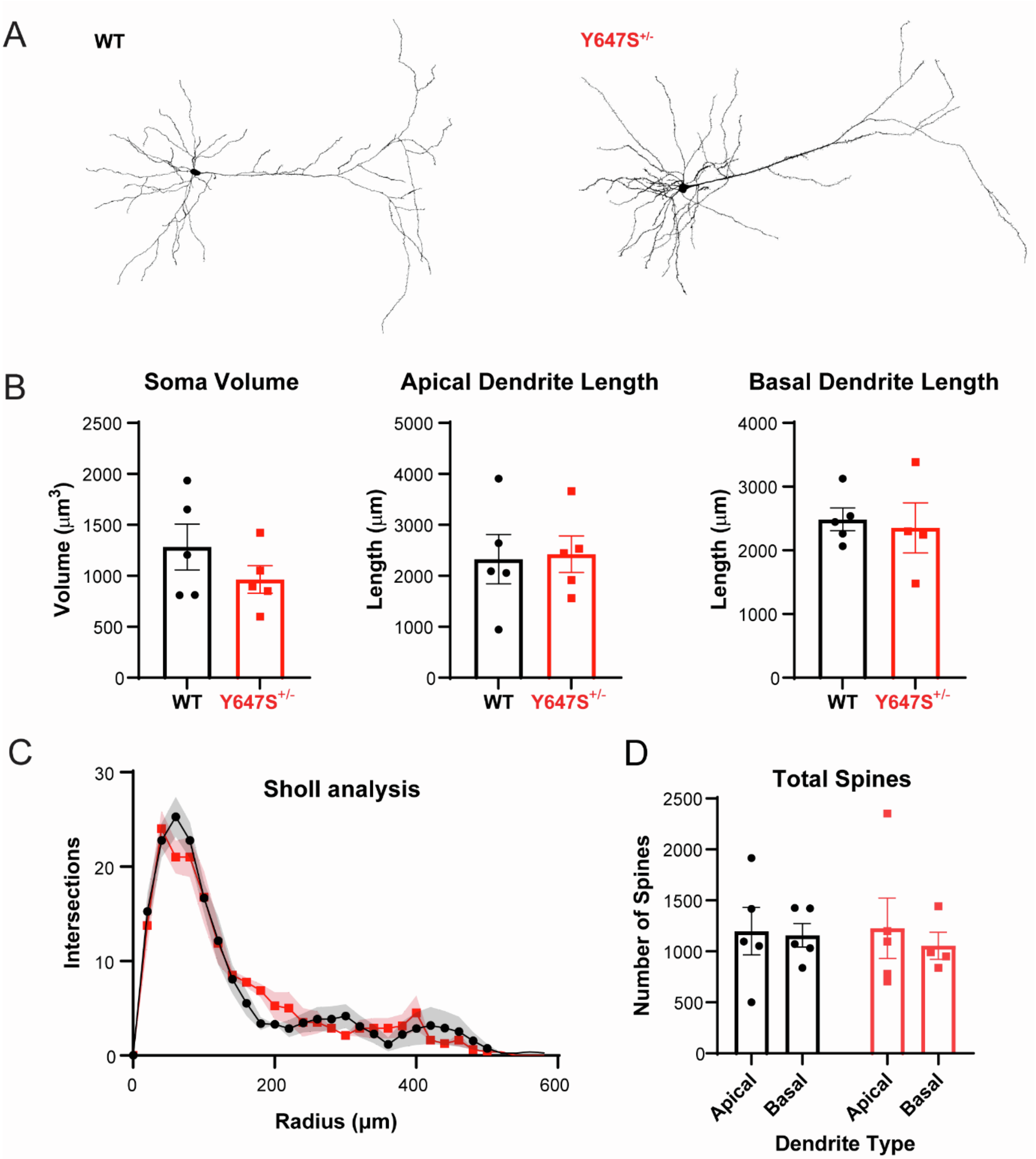

Supp Fig S3. Morphological properties of layer 5 pyramidal neurons are unaltered in Grin1 Y647S+/- mice. A, Example traces of layer 5 neurons from wildtype (WT) and Grin1 Y647S+/- mice. **B,** Soma volume, apical, and basal dendrite length are not significantly different between wildtype and Grin1 Y647S+/- mice. Each dot represents the average value per mouse, 1-3 neurons were imaged per mouse. **C,** Scholl analysis quantifying number of dendritic intersections at consecutive intervals from the soma. Shading represents SEM. Note, the SEM is too small relative to the size of the points and is not visible for certain points. **D,** Total number of spines in apical and basal dendrites. **(related to Fig 3)**

Supplemental Figure S4

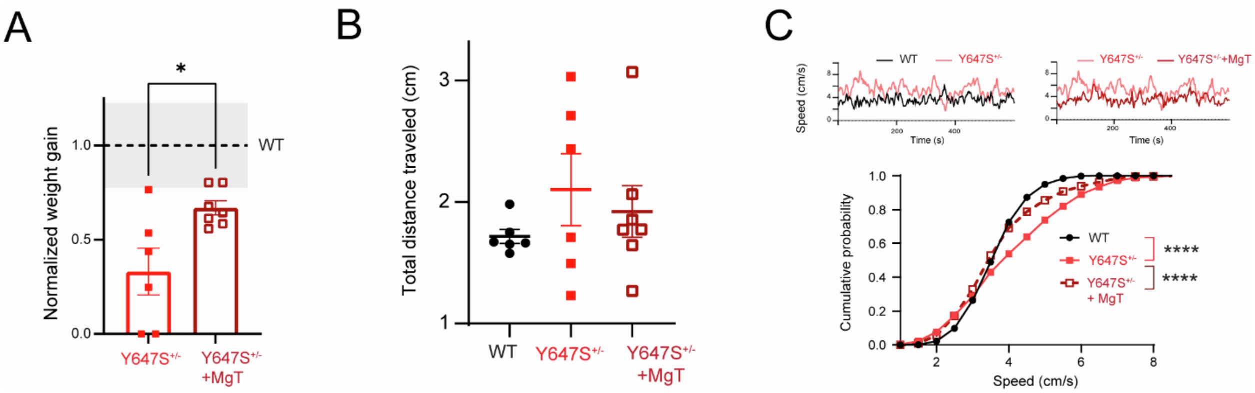

Supp Fig S4. Magnesium threonate improves weight gain and restores normal running velocity in *Grin1* Y647S$^{+/-}$ mice at 7 weeks of treatment. A, Normalized weight gain is significantly reduced in Y647S$^{+/-}$ compared to WT ($t_{(10)} = 2.45$, $P = 0.034$), but is improved in MgT treated Y647S$^{+/-}$ mice compared to untreated controls ($t_{(11)} = 2.77$, $P = 0.018$, $^*P < 0.05$). Standard error in WT weight gain is shown as the shaded grey rectangle. **B,** Total distance traveled in 10 minutes is not significantly different across WT, Y647S$^{+/-}$, and Y647S$^{+/-}$ +MgT (One-way ANOVA: F (2, 16) = 0.77, P = 0.48) but is more variable in Y647S$^{+/-}$ mice (Brown-Forsythe test: $F_{(2, 16)} = 4.23$, $P = 0.034$). **C,** Velocity profile comparing example WT and Y647S$^{+/-}$ mice (Top left), and the same Y647S$^{+/-}$ with an MgT-treated Y647S$^{+/-}$ mouse (Top right). Cumulative distribution of velocity in WT, Y647S$^{+/-}$, and Y647S$^{+/-}$+MgT mice (WT vs Y647S$^{+/-}$: $P < 10^{-4}$, Y647S$^{+/-}$ vs Y647S$^{+/-}$ + MgT: $P < 10^{-4}$, WT vs Y647S$^{+/-}$ + MgT: $P = 0.47$, Dunn's post hoc). $^{****}P < 10^{-4}$. **(related to Fig 7)**
